# Combination of* Ligusticum Chuanxiong* and* Radix Paeonia* Promotes Angiogenesis in Ischemic Myocardium through Notch Signalling and Mobilization of Stem Cells

**DOI:** 10.1155/2019/7912402

**Published:** 2019-02-17

**Authors:** Wei-Li Shi, Jun Zhao, Rong Yuan, Yan Lu, Qi-Qi Xin, Yu Liu, Wei-Hong Cong, Ke-Ji Chen

**Affiliations:** ^1^Laboratory of Cardiovascular Diseases, Xiyuan Hospital, China Academy of Chinese Medical Sciences, Beijing, 100091, China; ^2^TCM Department, The Affiliated Hospital of Qingdao University, Qingdao Shandong (266003), China; ^3^Graduate School, Beijing University of Chinese Medicine, Beijing 100029, China; ^4^Department of Neurology, Xiyuan Hospital, China Academy of Chinese Medical Sciences, Beijing, 100091, China; ^5^Pharmacy Department, Xiyuan Hospital, China Academy of Chinese Medical Sciences, Beijing, 100091, China

## Abstract

**Objective:**

To study the cardioprotective mechanism by which the combination of Chuanxiong (CX) and Chishao (CS) promotes angiogenesis.

**Methods:**

Myocardial infarction (MI) mouse models were induced by ligation of the left anterior descending coronary artery. The effects on cardiac function were evaluated in the perindopril tert-butylamine group (PB group) (3 mg/kg/d), CX group (55 mg/kg/d), CS group (55 mg/kg/d), and CX and CS combination (CX-CS) group (27.5 mg/kg/d CX plus 27.5 mg/kg/d CS). RO4929097, an inhibitor of Notch *γ* secretase, was used (10 mg/kg/d) to explore the role of Notch signalling in the CX-CS-induced promotion of angiogenesis in the myocardial infarcted border zone (IBZ). The left ventricular ejection fraction (LVEF) and percentage of MI area were evaluated with animal ultrasound and Masson staining. The average optical densities (AODs) of CD31 and vWF in the myocardial IBZ were detected by immunofluorescence. Angiogenesis-related proteins including hypoxia-inducible factor 1-alpha (HIF-1*α*), fibroblast growth factor receptor 1 (FGFR-1), Notch1 and Notch intracellular domain (NICD), and stem cell mobilization-related proteins including stromal cell-derived factor 1 (SDF-1), C-X-C chemokine receptor type 4 (CXCR-4), and cardiotrophin1 were detected by western blot analysis.

**Results:**

Compared with the model group, the CX-CS and PB groups both showed markedly improved LVEF and decreased percentage of MI area after 21 days of treatment. Although the CX group and CS group showed increased LVEF and decreased MI areas compared with the model group, the difference was not significant. The AOD of CD31 in the IBZ in both the model and the CX-CS-I group was markedly reduced compared with that in the sham group. CX-CS significantly increased the CD31 AOD in the IBZ and decreased the AODs of CD31 and vWF in the infarct zone compared with those in the model group. The expression of HIF-1*α* in both the model group and the CX-CS group was higher than that in the sham group. Compared with the model group, the expression of FGFR-1, SDF-1, cardiotrophin1, Notch1, and NICD was increased in the CX-CS group. Notch1 and NICD expression in the CX-CS-I group was reduced compared with that in the CX-CS group.

**Conclusions:**

The combination of CX and CS protected cardiomyocytes in the IBZ better than CX or CS alone. The mechanism by which CX-CS protects ischemic myocardium may be related to the proangiogenesis effect of CX-CS exerted through Notch signalling and the mobilization of stem cells to the IBZ.

## 1. Introduction

Cardiovascular disease, especially ischemic heart disease, remains the leading cause of mortality worldwide [1]. Although lots of patients suffering from myocardial infarction (MI) benefit from chemical medicine and the popularity of percutaneous coronary intervention (PCI), there is still a significant population of patients unable to receive effective treatment because of microvascular lesions, postoperative restenosis, and diffuse lesions. Therefore, exploration of novel therapeutic strategies to relieve ischaemia and preserve cardiac function is still necessary [2]. Traditional Chinese medicines (TCMs), especially the herbs for activating blood circulation and removing blood stasis (ABCRS), may provide such patients with an effective therapeutic option.

Modern research has confirmed that ABCRS herbs can improve haemodynamics, reduce inflammation, prevent platelet aggregation, and improve blood supply in ischemic myocardium to protect myocardial function [3–7]. Xuefu Zhuyu Decoction (XFZYD), created by Wang Qingren, is a classic ABCRS herbal prescription. There are five other famous prescriptions with similar effectiveness in Wang's book named* Yilin Gaicuo*. We found that* Ligusticum Chuanxiong* (family Apiaceae, also known as Chuanxiong, CX) and Red* Paeonia lactiflora Rubra* (family Paeoniaceae, also known as Chishao, CS) are both present in the abovementioned five prescriptions, except Shentong Zhuyu Decoction. In the six prescriptions from* Yilin Gaicuo*, the ratio of CX to CS is close to 1:1 (Table 1).

Restoration of blood flow in ischemic myocardium is pivotal to the treatment of ischemic heart disease. With the advancement of the concept of therapeutic angiogenesis, increasing numbers of studies have proved that angiogenesis effectively improves the blood supply of ischemic myocardium [8]. Angiogenesis is a complex process that forms new blood vessels from pre-existing vessels through budding. In contrast, vasculogenesis is the* de novo* formation of blood vessels [9–11]. Proangiogenic factors, such as vascular endothelial growth factor (VEGF) and basic fibroblast growth factor (bFGF), can markedly promote the proliferation of collateral vessels in ischemic areas to reduce infarct size [12, 13]. However, the clinical benefit of gene therapy remains controversial [14]. In addition, gene therapy faces some challenges, including the Janus phenomenon, the limited duration of effectiveness, the promotion of tumour growth, and other safety problems [8, 11, 15]. In addition to growth factors, stem cells also contribute to the formation of new vessels. It has been proved that stem cells secrete soluble paracrine factors and are able to differentiate into endothelial cells [16–19].

The complexity of TCMs is a disadvantage in terms of their worldwide promotion, but it is also an advantage for providing beneficial effects. Studies have shown that ABCRS herbs promote angiogenesis. For example, XFZYD promotes tube formation of human microvascular endothelial cells and mobilizes endothelial progenitor cells to accelerate angiogenesis [20–22]. Xiongshao Capsule consists of the total phenol of CX and the total glucoside of CS. A multicentre randomized double-blind placebo-controlled trial suggested that Xiongshao Capsule prevented restenosis after PCI [23]. The mechanism may be related to increased atherosclerotic plaque stability through the inhibition of platelet aggregation and the reduction in the levels of triglyceride and low-density lipoprotein cholesterol [24, 25]. The results suggested that Xiongshao Capsule could also promote angiogenesis of endothelial cells by upregulating the expression of bFGF and VEGF [26]. It was reported that 1 *μ*mol/L tetramethylpyrazine hydrochloride and 10 *μ*mol/L paeoniflorin synergistically promoted the angiogenesis in Tg(*fli-1a:EGFP)y1* zebrafish, and tetramethylpyrazine and paeoniflorin are the active ingredients of CX and CS, respectively [27].

TCM herb pairs are relatively fixed combinations of two herbs that are commonly used for certain pathological symptom(s) in clinical practice. Herb pairs are also the basic form of applied compatibility of TCM, which has not been paid enough attention to yet. The combination of CX and CS is a commonly used herb pair for cardiovascular diseases. In this study, the protective effects and mechanism of the CX-CS herb pair on ischemic myocardium were studied in an MI mouse model.

## 2. Materials and Methods

### 2.1. Preparation of CX and CS

Both CX and CS were purchased from Baicao Kangshen Co., Ltd. (Hebei, China), with quality report numbers C17121378 (CX) and C17121379 (CS). The decoctions were prepared by the Pharmacy Department of Xiyuan Hospital, China Academy of Chinese Medical Sciences (CACMS). CX and CS (300 g of each) were boiled separately after soaking in 2400 ml of pure water for 30 minutes. The first decoction lasted 1.5 h, and the second lasted 1 h. The filtrates were merged after two decoctions. Then, the merged filtrate was concentrated until 150 ml remained. Ultimately, an extract of CX and CS was obtained at a concentration of 2 g/ml.

### 2.2. Animals and MI Model Establishment

One hundred twenty specific pathogen-free (SPF) male C57BL/6 mice, weighing 20-25 g, were supplied by Vital River Laboratory Animal Technology Co., Ltd. (Beijing, China). Mice were kept under SPF environmental conditions with a 12 h light-dark cycle at 26 ± 1°C and 45 ± 10% humidity for 7 days before experiments. All animal procedures and protocols were performed in accordance with the Guide for the Care and Use of Laboratory Animals (NIH publication, 85-23, revised 1996) and were reviewed and approved by the Animal Research Committee at Xiyuan Hospital, CACMS. The MI mouse model was established by ligation of the left anterior descending coronary artery after mice were anaesthetized with 4% chloral hydrate injection, as previously described [28]. After thoracic hair was shaved, mice were sterilized with iodophor and alcohol in turn. A small animal ventilator was opened, and tracheal intubation was performed to assist with ventilation. The anterior chest skin was cut to separate the fascia and muscle layers and expose the ribs. The third rib was cut with a small pair of scissors to expose the heart. A needle was inserted and ligated in the middle of the right lower edge of left atrial appendage and the pulmonary conus. Sham-operated mice were fitted with sutures but were not subjected to ligation. After successful operation, the mice were placed on a mat to keep their body temperature at 37.0 ± 0.5°C until they recovered full consciousness. The success rate of operation was more than 70%, and 90 live mice were used in the study, including 20 sham-operated mice and 70 MI model mice.

### 2.3. Animal Grouping and Drug Administration

Two batches of mice were adopted in this study. The first batch of mice was divided into six groups, including the sham, model, perindopril tert-butylamine (3 mg/kg/d) (PB, 2010072, Servier, China), CX (55 mg/kg/d), CS (55 mg/kg/d), and CX-CS herb pair (27.5 mg/kg/d CX plus 27.5 mg/kg/d CS) groups, to determine the herbal medicine with the optimal effects on heart function and percentage of MI area [29, 30]. RO4929097 (s1575, Selleckchem, UK), an inhibitor of Notch *γ* secretase, was used to explore the role of the Notch/Notch intracellular domain (NICD) pathway in Chinese medicine-mediated promotion of angiogenesis in ischemic myocardium. The second batch of mice was divided into four groups, including the sham, model, CX-CS, and CX-CS plus RO4929097 (10 mg/kg/d) groups, to detect the effects of herb pair on the left ventricular ejection fractions (LVEFs) and average optical densities (AODs) of CD31 and vWF to determine the candidate relevant angiogenic mechanism. RO4929097 was formulated as a suspension in 1% carboxymethyl cellulose with 0.2% Tween 80 (C8621 and T8360, Solarbio, China) [31, 32]. Drugs were intragastrically administered to mice from the next day after model establishment once daily for 21 days. The shams and models were administered with equal volumes of physiological saline. All mice were allowed to move and take food freely before sacrificed on the 22nd day. Serum was separated for an enzyme-linked immunosorbent assay (ELISA). Hearts were removed on ice and cut into two parts beneath the ligature for histopathologic observation fixed in 4% paraformaldehyde and for western blot examination stored at −80°C.

### 2.4. Ultrasonic Assessment of LVEF

The LVEFs of mice were detected with an ultrasound imaging instrument for small animals (Vevo 2100, VisualSonics, Canada). Mice were anaesthetized with 4% chloral hydrate. An MS-250 probe was placed on the left chest and directed to the right side to achieve a long-axis view of the left ventricle. The mean values of three consecutive cardiac cycles were measured in each mouse, and the LVEF in each group was calculated.

### 2.5. Evaluation of Percentage of MI area by Masson Staining

Hearts were fixed in 4% paraformaldehyde for 12 h and then dehydrated in different concentrations of alcohol. Dehydrated samples were embedded in paraffin, sectioned, and serially stained with Masson (G1006, Servicebio, China). Myocardial samples after Masson staining were scanned with a digital slice scanner (Panoramic MIDI, 3DHISTECH, Hungary). The pixels of infarcted myocardium, characterized by a blue colour, and the pixels of normal myocardium, characterized by a red colour, were calculated by ImageJ 1.52a (Wayne Rasband, National Institutes of health, USA). The percentage of MI area was calculated as blue pixels/(blue plus red pixels) multiplied by 100%.

### 2.6. Immunofluorescence of CD31 and vWF

Tissue sections obtained after sectioning as described above were incubated with antibodies of CD31 (GB12063, Servicebio, China) and vWF (GB11020, Servicebio, China) and 4′,6-diamidino-2-phenylindole (DAPI, G1012, Servicebio, China) in sequence. Five photos (200X) of the infarcted border zone (IBZ) and MI area were obtained by microscopy (DMIL-RF1, Leica, Germany). The integrated density and area of CD31 and vWF were calculated using ImageJ. The AODs of CD31 and vWF were calculated as the integrated density/area multiplied by 100%.

### 2.7. Measurement of VEGF and bFGF

Serum VEGF (KE10009, Proteintech, USA) and bFGF (ab100670, Abcam, UK) were detected by ELISA according to the manufacturer's instructions. Optical density was analysed with a microplate reader (Epoch2, Biotek, USA).

### 2.8. Quantification of HIF-1*α*, FGFR-1, Notch1/NICD, SDF-1, CXCR-4, and Cardiotrophin1 in Samples by Western Blot Analysis

Precooled radioimmunoprecipitation assay (RIPA) protein extraction reagent was mixed with protease inhibitor. The tissues were homogenized with an electric homogenizer, incubated on ice for twenty minutes, and centrifuged at 14,000 × g. The protein concentrations of the samples were determined with the bicinchoninic acid (BCA) method and adjusted to the lowest concentration with RIPA buffer. The protein obtained from all tissues was denatured for five minutes at 95°C. An SDS-polyacrylamide gel was prepared, and the samples were loaded onto the gel, electrophoresed, and transferred onto polyvinylidene fluoride (PVDF) membranes. Then, 5% bovine serum albumin was used for blocking. After incubation with primary antibodies against GAPDH (5174, CST, USA), hypoxia-inducible factor 1-alpha (HIF-1*α*) (36169, CST, USA), FGF Receptor 1 (FGFR-1) (9740, CST, USA), Notch1 (3608, CST, USA), cleaved Notch1 (also named NICD) (4147s, CST), stromal cell-derived factor-1 (SDF-1) (3740, CST, USA), C-X-C chemokine receptor 4 (CXCR-4) (60042-1-1g, Proteintech, USA), and cardiotrophin1 (YT0638, ImmunoWay, USA) at 4°C, the membranes were incubated with a secondary antibody (115-035-003, Jackson, USA) at room temperature for two hours. Finally, the membranes were imaged with a Gel Imager (ChemicDoc XRS+, Bio-Rad, USA) and analysed with ImageJ.

### 2.9. Statistical Analysis

Statistical analyses were performed using GraphPad Prism 6 (GraphPad Software Inc., San Diego, CA, USA). Variables with normal distributions are shown as $\overset{-}{x}$  ± SEM. A one-way ANOVA with the least significant difference (LSD)* post hoc* test was performed for analyses among groups. A* p-*value less than 0.05 was considered statistically significant.

## 3. Results

### 3.1. Comparison of the Effects of CX, CS, and CX-CS on Cardiac Function

#### 3.1.1. LVEF Comparison

**Figure 1:**
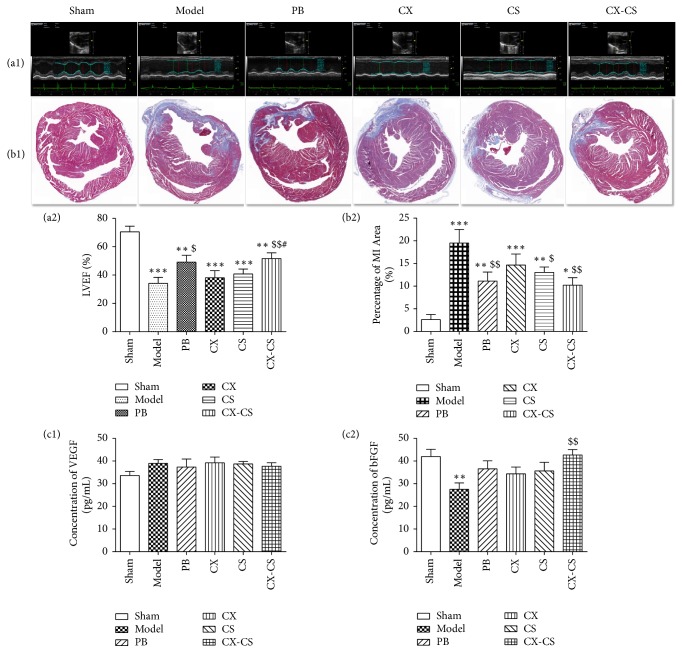
Comparison of LVEF, percentage of MI area, and serum level of VEGF and bFGF in the sham, model, PB, CX, CS, and CX-CS groups. LVEF of mice was assessed using ultrasound imaging instrument. Percentage of MI area was detected after Masson staining and growth factors, including VEGF and bFGF, was examined by Elisa. The results suggested CX-CS herb pair improved cardiac function more obviously than CX or CS group. ^*∗*^*P*<0.05, ^*∗∗*^*P*<0.01, ^*∗∗∗*^*P*<0.001 vs. sham group; ^$^*P*<0.05, and ^$$^*P*<0.01 vs. model group; ^#^*P*<0.05 vs. CX group. n=7.

Left ventricular contractive function decreased after the ligation of the left anterior descending coronary artery accompanied by abnormal motion of the ventricular wall. The LVEF of the model group decreased significantly compared with that of the sham group (*P*<0.001). After treatment administration, LVEF increased markedly in both PB group and the CX-CS groups (*P*<0.05 or* P*<0.01) compared with that of the model group. There was no significant difference between the model group and the CX group (*P*>0.05) or the CS group (*P*>0.05) (Figures 1(a1) and 1(a2)).

#### 3.1.2. Evaluation of the MI Area Ratio

Myocardial cells in the noninfarct zone were dyed red, and the infarct zone was dyed blue after Masson staining. Infarct tissue was characterized by collagen fibers and inflammatory cells. Compared with the sham group, the percentage of MI size/left ventricular area in the model and treatment groups was markedly increased. After treatment, the percentages of MI size/left ventricular area in the PB group, CS group, and CX-CS group were decreased significantly compared with that in the model group (*P*<0.05 or* P*<0.01) (Figures 1(b1) and 1(b2)).

#### 3.1.3. Levels of Serum VEGF and bFGF

Serum bFGF level of the models was significantly decreased compared with that of the shams (*P<*0.01). After treatment, bFGF levels in the PB, CX, CS, and CX-CS groups were all enhanced, and levels in mice of the CX-CS group were markedly increased compared with those in the model group (*P<*0.01) (Figure 1(c2)). There was no significant difference in VEGF levels among all groups (*P>*0.05) (Figure 1(c1)). The comparison of LVEF, percentage of MI area, and levels of growth factor and inflammatory factor in the CX, CS, and CX-CS groups indicated that the CX-CS herb pair improved cardiac function more obviously better than CX or CS alone.

### 3.2. Cardioprotective Mechanisms of the CX-CS Herb Pair

#### 3.2.1. LVEF and MI Area Measurements and Histopathological Observation after Inhibition of Notch *γ* Secretase

**Figure 2:**
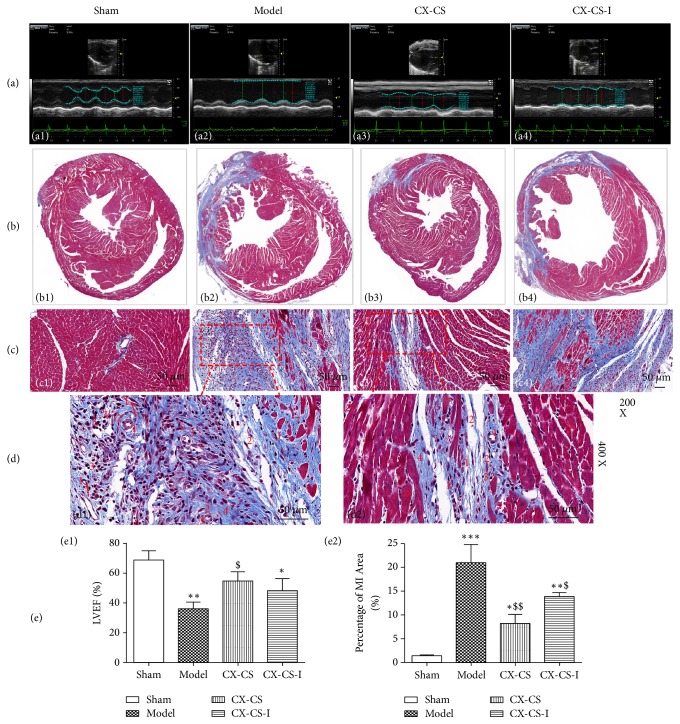
LVEF, percentage of MI area, and histopathological observations after inhibition of Notch *γ* secretase. (a) Effect of Notch inhibition on LVEF. (b) Effect of Notch inhibition on percentage of MI area. (c) Histopathological observations after inhibition of Notch. (d) Granulation observation. (e) Statistical analysis of LVEF and percentage of MI area. No. 1 capillaries (arrows point to endothelial cells), No. 2 fibroblast, No. 3 inflammatory cell, No. 4 collagen fibre, No. 5 normal cardiac myocytes, No. 6 hypertrophic cardiomyocyte, and No. 7 dissolution of cardiac myocyte. ^*∗*^*P*<0.05, ^*∗∗*^*P*<0.01, ^*∗∗∗*^*P*<0.001 vs. sham group; ^$^*P*<0.05, and ^$$^*P*<0.01 vs. model group. n=6.

Cleavage of the Notch receptor ultimately releases the NICD, which translocates to the nucleus and initiates gene transcription. When *γ* secretase activity is inhibited by RO4929097, NICD release is blocked. In this study, the LVEF of the CX-CS group was markedly improved compared with that of the model group, and there was no significant difference in LVEF between the CX-CS-I and model groups (Figures 2(a) and 2(e1)). Compared with the model group, the percentage of MI area in the CX-CS and CX-CS-I groups was decreased (*P*<0.01 or* P*<0.05) (Figures 2(b) and 2(e2)). This finding indicated that the Notch/NICD pathway may play an important role in CX-CS-mediated protection of cardiac function after MI although it might not be the only pathway involved. Masson staining showed that the myocardial cells were arranged regularly and that the myocardial fibre structure was clear with no or only a small number of inflammatory cells in the noninfarcted area and sham group (Figures 2(c1), 2(c3), and 2(d2)). In the infarcted area, the myocardial muscle was ruptured and dissolved, accompanied by a large amount of disordered fibrous proliferation. In addition, the inflammatory reaction was obvious, characterized by a large number of infiltrating inflammatory cells (Figures 2(c2) and 2(d1)).

After MI, the myocardium underwent coagulation necrosis and dissolved gradually. The granulation tissue formed to absorb and replace the necrotic tissue subsequently, which consisted of newborn capillaries, proliferating fibroblasts, and inflammatory cells (Figures 2(d1) and 2(d2)). Endothelial cells proliferated to form solid cell lines and dilated capillaries (Figures 2(d1) and 2(d2), No.1). There were many new fibroblasts around these capillaries. In addition, there was much exudate, and there were many inflammatory cells (Figures 2(d1) and 2(d2), No. 2-3), especially macrophages. In the model group and the CX-CS-I group, the inflammatory reaction was stronger than that of the CX-CS group.

#### 3.2.2. AODs of CD31 and vWF in the IBZ

**Figure 3:**
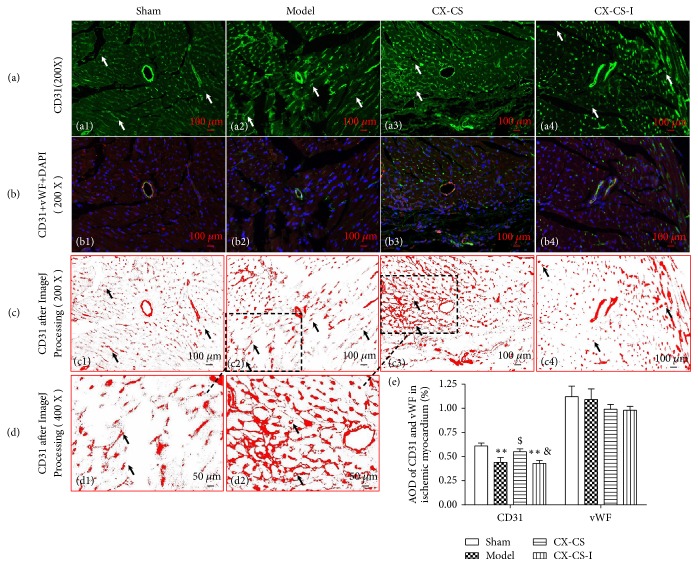
AODs of CD31 and vWF in the IBZ. (a) Immunofluorescence staining of CD31 in the IBZ. (b) Merged images of CD31, vWF, and DAPI. (c and d) Images processed by ImageJ after immunofluorescence staining of CD31. (e) AOD analysis of CD31 and vWF. The arrows point to angiogenesis or endothelial cells. ^*∗*^*P*<0.05; ^*∗∗*^*P*<0.01* vs.* sham group; ^$^*P*<0.05 vs. model group; ^&^*P*<0.05* vs.* CX-CS group. n=6.

CD31 and vWF are specific marker proteins for vascular endothelial cells. After immunofluorescence staining with CD31 (green), vWF (red), and DAPI (blue), the vascular morphology was observed. Since it was difficult to count vascular numbers because the vascular lumina were small or fuzzy, the AODs of CD31 and vWF were used to evaluate the density of angiogenesis. The staining showed newborn vessels characterized by green points and nuclei characterized by blue staining distributed evenly (Figures 3(a1) and 3(b1)) in the shams. After processing with ImageJ, the vessels became clearer. Angiogenesis with lumina less than 20 *μ*m could be observed. The vascular lumen surrounded by an endothelial cell monolayer was also clearly visible (Figures 3(c) and 3(d)). In the model group, nuclei and angiogenesis became irregular or uneven because compensatory hypertrophy or partial dissolution of ischemic cardiomyocytes resulted from infarction (Figures 3(a2), 3(b2), 3(c2), and 3(d1)). Compared with the sham group, the CD31 AODs in mice of the model and CX-CS-I groups were markedly decreased (*P<*0.001), while significantly increasing in the CX-CS group compared with that in the models (*P<*0.05) (Figure 3(e)).

The capillary network formed in the IBZ to improve the blood supply to the ischemic myocardium, and the nuclei were denser compared the CX-CS-treated mice with the models (Figures 3(a3), 3(b3), 3(c3), and 3(d2)). After intervention with CX-CS plus inhibitor, CD31 AODs decreased compared with that of CX-CS. This result indicated that the Notch pathway may play a role in the promotion of IBZ angiogenesis mediated by the CX-CS herb pair.

#### 3.2.3. AODs of CD31 and vWF in the MI Area

**Figure 4:**
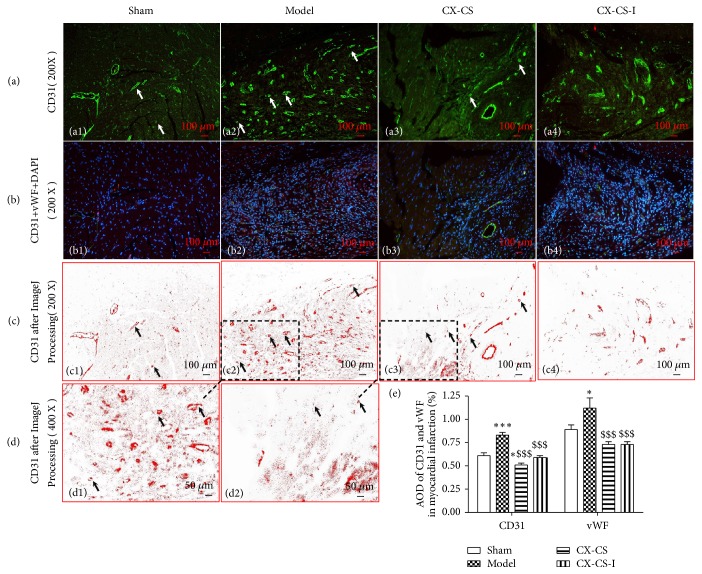
AODs of CD31 and vWF in MI area. (a) Immunofluorescence staining of CD31 in the MI area. (b) Merged images of CD31, vWF, and DAPI. (c and d) Images processed by ImageJ after immunofluorescence staining of CD31. (e) AOD analysis of CD31 and vWF. The arrows point to angiogenesis or endothelial cells. ^*∗*^*P*<0.05; ^*∗∗∗*^*P*<0.001* vs.* sham group; ^$$$^*P*<0.001* vs*. model group. n=6.

Necrotic tissue in the MI area could not be completely dissolved, absorbed, or discharged. Granulation tissue formed and was composed of new capillaries and fibroblasts. The pathological process in the MI area was not the same as that in the IBZ. In the model group, the AODs of both CD31 and vWF were significantly increased compared with those in the sham group (*P<*0.05 or* P*<0.001). Nuclei were denser in the model group than in the sham group because granulation formation was characterized by angiogenesis, the proliferation of fibroblasts, and the infiltration of inflammatory cells (Figures 4(a2), 4(b2), 4(c2), and 4(d1)). The AODs of CD31 and vWF were decreased in the CX-CS group and CX-CS-I group compared with those in the model group (*P<*0.001) (Figure 4(e)).

#### 3.2.4. Serum VEGF and bFGF

**Figure 5:**
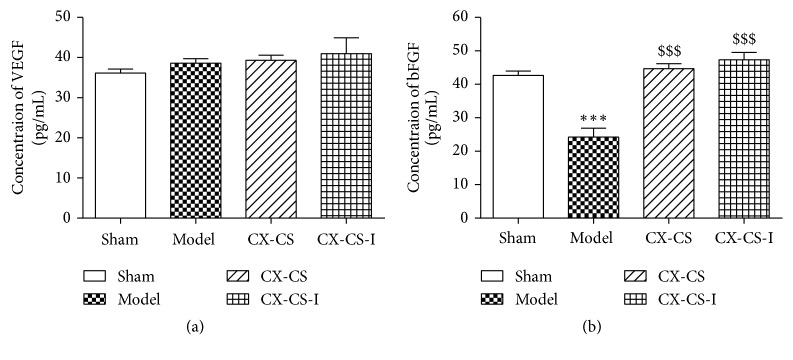
Serum VEGF and bFGF. There was no significant difference in VEGF levels among the groups (*P*>0.05). The bFGF levels in the CX-CS and CX-CS-I groups increased markedly compared with model group (*P*<0.001). ^*∗∗∗*^*P*<0.001* vs.* sham group; ^$$$^*P*<0.001* vs.* model group. n=6.

In this study, serum growth factors were measured. The bFGF level of the models was significantly decreased compared with that of the shams (*P<*0.001). Compared with that of models, the bFGF levels in the CX-CS group and the CX-CS-I group increased markedly (*P<*0.001) (Figure 5(b)). There was no significant difference in VEGF levels among the groups (*P>*0.05) (Figure 5(a)).

#### 3.2.5. Expression of HIF-1*α*, FGFR-1, SDF-1, CXCR-4, and Cardiotrophin1

**Figure 6:**
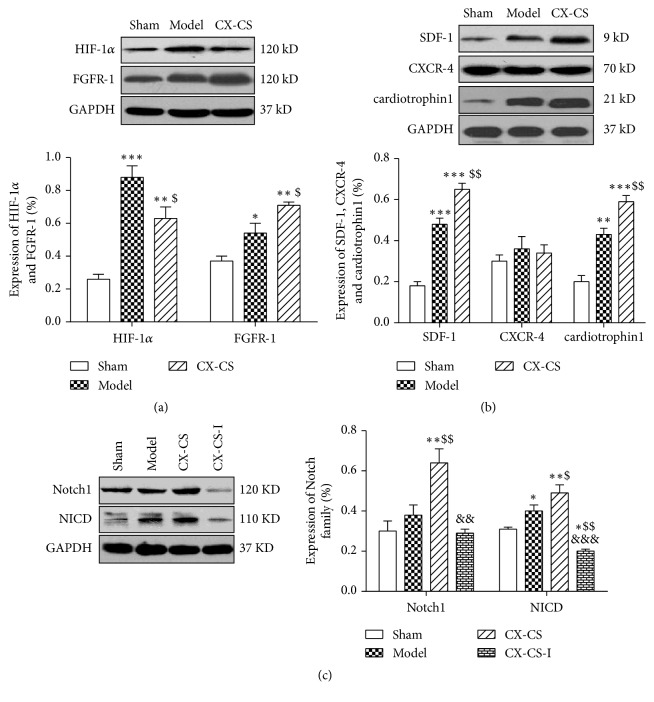
Expression of angiogenesis and stem cell mobilization proteins. (a) Protein levels of HIF-1*α* and FGFR-1. (b) Protein levels of SDF-1, CXCR-4, and cardiotrophin1. (c) Protein levels of Notch1 and NICD. ^*∗*^*P*<0.05, ^*∗∗*^*P*<0.01, and ^*∗∗∗*^*P*<0.001* vs.* sham group; ^$^*P*<0.05, ^$$^*P*<0.01* vs.* model group; ^&&^*P*<0.01* vs.* CX-CS group. n=3.

Angiogenesis involves a series of biological processes. Stem cell mobilization also participates in angiogenesis. Here, we examined the expression of HIF-1*α* and FGFR-1. SDF-1, CXCR-4, and cardiotrophin1 were also measured to study the effect of CX-CS on stem cell mobilization. The expression of HIF-1*α* in the model group and the CX-CS group was significantly increased compared with that in the sham group (*P<*0.001 or* P<*0.01). The expression of HIF-1*α* in CX-CS group decreased (*P<*0.05), while FGFR-1 expression increased (Figure 6(a)). There was no difference in the expression of CXCR-4, a protein involved in stem cell mobilization, in any of the groups (*P>*0.05). Compared with the shams, the expression of SDF-1 and cardiotrophin1 was markedly increased in the model group and the CX-CS group (*P<*0.001 or* P<*0.01). The expression of SDF-1 and cardiotrophin1 in the CX-CS group was significantly increased compared with that in the model group (*P<*0.01) (Figure 6(b)).

#### 3.2.6. Expression of Notch1 and NICD

The Notch family is involved in angiogenesis and participates in the differentiation of stem cells. In this study, RO4929097 was used to explore the involvement of the Notch/NICD pathway in the effects of CX-CS on angiogenesis. CX-CS increased the expression of Notch1 and NICD compared with that of model group (*P<*0.05). After CX-CS-I treatment, the expression of Notch1 and NICD was decreased compared with that in the CX-CS group (*P<*0.01 or* P<*0.001) (Figure 6(c)).

## 4. Discussion 

Although the CX-CS herb pair is commonly used for blood stasis syndrome and is effective for ischemic diseases, the cardioprotective mechanisms remain unclear. Our results demonstrate that treatment with the CX-CS herb pair significantly improved LVEF and reduced the percentage of MI area in mice compared with treatment with CX or CS alone. CX-CS also increased the AOD of CD31 in the IBZ, which suggested that the cardioprotective effect of CX-CS may relate to the promotion of angiogenesis. A key issue regarding ischaemia-induced angiogenesis is whether the new capillaries are effective in increasing blood supply. Whether Chinese ABCRS herbs act preferentially in specific tissues is a topic deserving further research. As shown in the results, more granulation tissues formed in the model group, which was also characterized by new blood vessels. It suggested that the new vessels in the infarcted area may only promote the maturation of granulation tissue to accelerate the fibrosis of infarcted myocardium, resulting in less blood supply for ischemic tissue.

Angiogenesis is stimulated mainly by tissue hypoxia via the HIF-1*α* activation [33]. HIF-1*α* activates the transcription of numerous genes, including VEGF, VEGF receptor flt-1, and bFGF [34, 35]. BFGF, a potent angiogenic factor, stimulates the proliferation of fibroblasts and endothelial cells that are involved in angiogenesis and the development of granulation tissue. HIF-1*α* and FGFR-1 expression in both the CX-CS group and the model group was markedly increased compared with that in the sham group, and HIF-1*α* expression was decreased in the CX-CS group compared with that in the model group. The expression of HIF-1*α* is strictly influenced by oxygen concentration. Under hypoxia, the ubiquitination and degradation of HIF-1*α* are inhibited, and the resulting accumulation of HIF-1*α* in the cytoplasm stimulates multiple angiogenic factors, including bFGF [36, 37]. HIF-1*α* expression in the CX-CS group decreased, which suggested after the treatment of CX-CS herb pair that oxygen concentration in the ischemic myocardium has been increased.

Stem cell-based therapies provide promising therapeutic potential for the formation of new blood vessels in ischaemia. It has been proven that SDF-1 greatly contributes to mobilization and recruits stem cells to ischemic tissue by combining with CXCR-4 [38, 39]. SDF-1 not only facilitates the homing of engrafted bone mesenchymal stem cells (MSCs) but also increases vascular density and induces angiogenesis in ischaemia [40]. Cardiotrophin1, a powerful cytokine promoting cell engraftment, preserves cardiac function in infarcted hearts by promoting the persistence and adhesion of bone MSCs [41]. It has also been shown that cardiotrophin1 promotes cardiac differentiation of stem cells [42, 43]. The results of this study demonstrated that CX-CS significantly enhanced SDF-1 and cardiotrophin1 expression, which suggested that stem cells may be mobilized to ischemic myocardium to protect injured tissue. However, the exact mechanism by which CX-CS regulates stem cells to promote angiogenesis in ischemic cardiomyocytes requires further study.

Notch signalling controls the angiogenic growth of the blood vessel network, the proliferation of endothelial cells, and the differentiation of arteries and veins [44]. There are four receptors (Notch1-4) and five ligands (DII1, DII3, DII4, Jaggedl, and Jagged2) in mammalian cells. Notch signalling occurs once a Notch ligand binds to its receptor, and the Notch receptor releases NICD from the cell membrane. Then, NICD translocates to the nucleus to regulate gene transcription, thereby influencing the proliferation and differentiation of cells [45]. Notch1/NICD is involved in the angiogenesis of ischemic tissue and favours stem cell differentiation into cardiomyocytes [46–48]. In this study, CX-CS upregulated Notch and NICD expression and no longer increased the NICD level after Notch *γ* secretase was inhibited, which suggested that Notch1/NICD signalling might play an important role in the CX-CS-mediated promotion of angiogenesis in ischemic myocardium. However, whether hypoxia influences Notch activation remains unclear.

## 5. Conclusion

In conclusion, our results reveal that the cardioprotective effect of the CX-CS herb pair on MI mice may depend on its proangiogenesis effect in the ischemic zone. Furthermore, both Notch signalling and stem cell mobilization may participate in the angiogenesis promoted by CX-CS. The results of this study help to deepen the understanding of ABCRS herbs and supply more evidence for the usefulness of CX-CS as a treatment or combination therapy for myocardial ischaemia-related diseases.

## Figures and Tables

**Table 1:** ABCRS herbal prescriptions in *Yilin Gaicuo*.

Name of prescription	CX (g)	CS (g)	CX:CS ratio
Xuefu Zhuyu Decoction	4.5	6	3:4
Tongqiao Huoxue Decoction	3	3	1:1
Gexia Zhuyu Decoction	6	6	1:1
Shaofu Zhuyu Decoction	6	6	1:1
Shentong Zhuyu Decoction	6	-	-
Buyang Huanwu Decoction	3	5	3:5

The doses of CX and CS were taken from the book of *Pharmacology of TCM Formulae* published by the China Press of TCM.

## Data Availability

The data used to support the findings of this study are available from the corresponding author upon request.
